# Is ADHD a sleep disorder? can adhd improve by treating the comorbid sleep disorder(S)? a research update

**DOI:** 10.1192/j.eurpsy.2021.1483

**Published:** 2021-08-13

**Authors:** J.J.S. Kooij

**Affiliations:** Psychiatry, Amsterdam UMC/VUmc, Amsterdam, Netherlands

**Keywords:** Treatment, Adult, ADHD, sleep disorders

## Abstract

**Introduction:**

Research has shown that ADHD and sleep disorders are intimately intertwined in the majority of patients in both childhood and adulthood. Circadian rhythm sleep disturbances, esp. the delayed sleep phase syndrome, as well as several other sleep disorders, such as Insomnia, Restless Legs, Periodic Limb Movement Disorder and Sleep apnea are associated with ADHD. With a prevalence rate of 80% of sleep disorders in adults with ADHD, the question not only is what is chicken and egg, but even if both conditions share a joint pathophysiology.

**Objectives:**

To investigate the consequences of this comorbid sleep disorders on severity of ADHD, mood and health, as well as to find evidence on improvement of ADHD by treatment of the sleep disorder(s).

**Methods:**

Recent research will be evaluated to formulate answers to these questions.

**Results:**

Sleep loss resulting from sleep disorders increases ADHD severity due to more impairment of cognition and memory as well as mood instability. Sleep loss in the longer term also leads to obesity, with negative consequences for health in general. First studies showing a decrease of ADHD symptoms by treatment of sleep disorders will be discussed.

**Conclusions:**

ADHD and sleep disorders come together in the majority of patients and need both assessment and treatment. Treatment of ADHD by improving sleep, is an intriguing research question with potential new treatment options.

